# Genetic Analysis of the Henry Mountains Bison Herd

**DOI:** 10.1371/journal.pone.0144239

**Published:** 2015-12-16

**Authors:** Dustin H. Ranglack, Lauren K. Dobson, Johan T. du Toit, James Derr

**Affiliations:** 1 Department of Wildland Resources and Ecology Center, Utah State University, Logan, Utah, United States of America; 2 Department of Veterinary Pathobiology, Texas A&M University, College Station, Texas, United States of America; 3 Department of Wildland Resources, Utah State University, Logan, Utah, United States of America; Embrapa, BRAZIL

## Abstract

Wild American plains bison (*Bison bison*) populations virtually disappeared in the late 1800s, with some remnant animals retained in what would become Yellowstone National Park and on private ranches. Some of these private bison were intentionally crossbred with cattle for commercial purposes. This forced hybridization resulted in both mitochondrial and nuclear introgression of cattle genes into some of the extant bison genome. As the private populations grew, excess animals, along with their history of cattle genetics, provided founders for newly established public bison populations. Of the US public bison herds, only those in Yellowstone and Wind Cave National Parks (YNP and WCNP) appear to be free of detectable levels of cattle introgression. However, a small free-ranging population (~350 animals) exists on public land, along with domestic cattle, in the Henry Mountains (HM) of southern Utah. This isolated bison herd originated from a founder group translocated from YNP in the 1940s. Using genetic samples from 129 individuals, we examined the genetic status of the HM population and found no evidence of mitochondrial or nuclear introgression of cattle genes. This new information confirms it is highly unlikely for free-living bison to crossbreed with cattle, and this disease-free HM bison herd is valuable for the long-term conservation of the species. This bison herd is a subpopulation of the YNP/WCNP/HM metapopulation, within which it can contribute significantly to national efforts to restore the American plains bison to more of its native range.

## Introduction

Once numbering in the millions, plains bison (*Bison bison)* populations across North America dramatically declined from over-harvesting to less than 100 wild bison by the late 1800s [1]. Private individuals led bison conservation by capturing and raising wild bison on private ranches [2,3]. By the late 1800s, bison and cattle were crossbred for commercial purposes, leading to both mitochondrial and nuclear introgression of cattle genes into much of the remaining bison populations. Wild bison persisted in small groups in Yellowstone National Park (YNP), USA, and Banff National Park, Canada. Of the ~500,000 bison in North America today, only ~20,000 are found in conservation herds while the others are all in private commercial livestock production herds [4,5]. ‘Conservation herds’ are defined as herds that are managed by federal or state/provincial governments or non-governmental organizations whose mission is nature conservation [6]. Whereas many private herds are raised for values other than livestock production (aesthetics, public viewing, conservation), they are always vulnerable to economic forces that could jeopardize the security of their conservation status.

**Fig 1:**
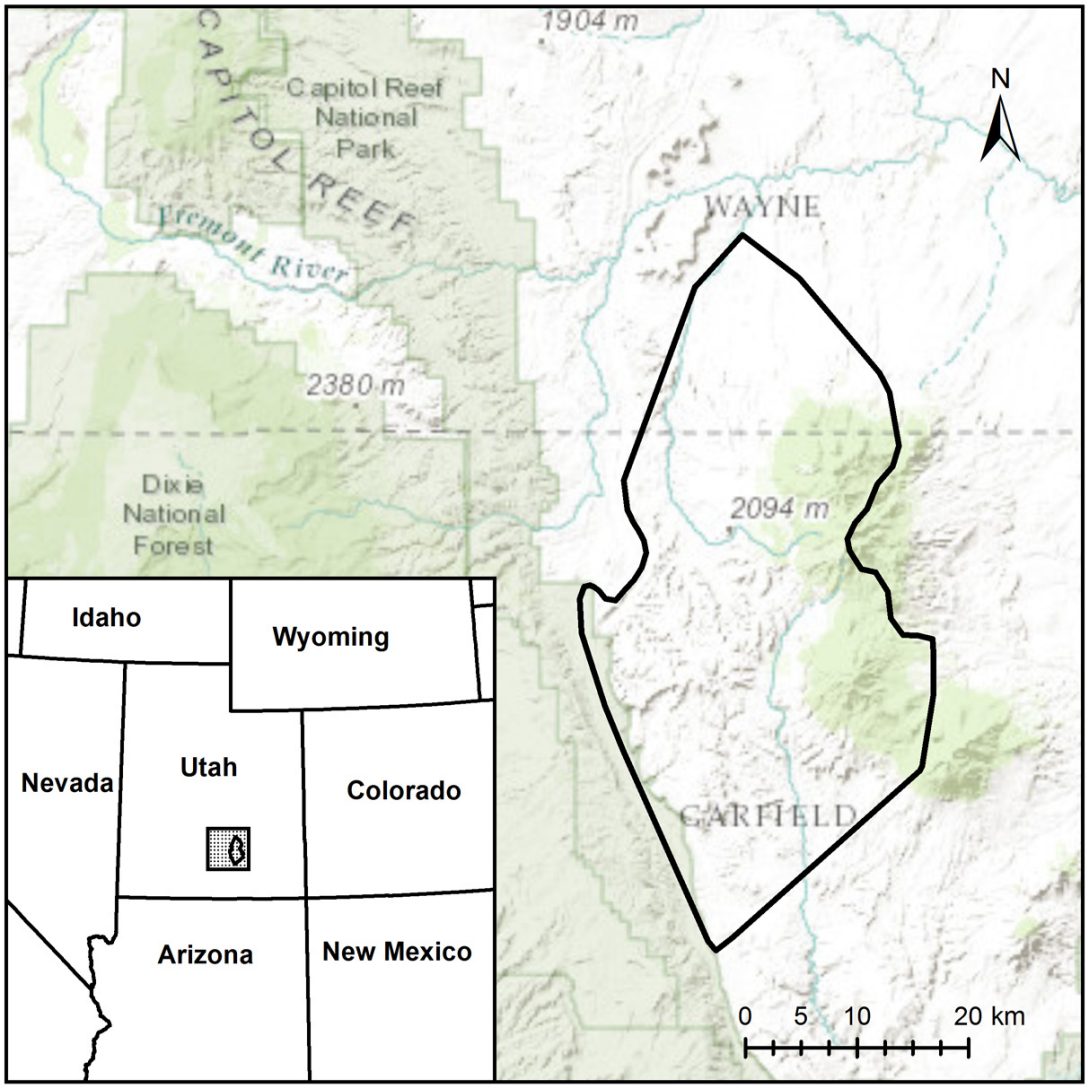
Henry Mountains. The location of the Henry Mountains in the state of Utah. Tissue (tail hair) samples for genetic analysis were collected from bison throughout the area enclosed by the black polygon.

Despite the relative successes of several conservation herds and the large number of commercial herds, bison have not fully recovered ecologically as a wildlife species [5,7]. Most conservation herds are small, isolated, and intensively managed within fences where they exist without natural predators [6]. While the number of conservation herds is growing, the total number of bison in conservation herds remains relatively constant [5]. Conflict exists between bison conservationists and livestock managers due to issues of competition, disease, and genetic introgression, leading to most bison herds being kept separate from cattle by fences and hazing practices [8]. Additionally, the residual effects of early bison-cattle hybridization efforts are documented in 6 of the 8 major federal bison herds, with Yellowstone and Wind Cave National Parks being the only federal conservation herds where cattle introgression has been screened for but not detected [9]. These two populations represent the largest bison conservation herds, yet, as we report here, the disease-free population in the Henry Mountains (HM) of southern Utah (Fig 1) also appears to be free of introgression by domestic cattle genes and thus represents a third such population of plains bison on public land in North America.

The HM bison herd is a closed population that was established in 1941 with 18 bison (3 bulls and 15 cows) from YNP. An additional 5 bulls were released in 1942 when some or all of the original bulls dispersed away the from herd [10,11]. The HM bison herd grew rapidly, reaching an all-time high in 2007 [12], and now numbers ~350 adults. The bison range over an area of ~125,000 ha. The population has been controlled primarily through sport hunting since 1960 using a combination of “cow only” and “hunter’s choice” tags in an escapement threshold harvesting scheme [13]. The HM area is primarily public land managed by the Bureau of Land Management (BLM), whereas the bison herd is managed by the Utah Division of Wildlife Resources. This herd is unique in that it is free-ranging, disease-free, huntable, and occurs with cattle on unfenced grazing allotments. Most previous research on the HM bison herd has focused on the potential for competition with cattle [13–19] whereas comparatively little attention has been paid to the genetic status of the herd [9,20–22]. Introgression of cattle genes has not been detected in HM bison and the known founders of the herd (from YNP) were assumed to be free of cattle genes, but until now sample sizes were too small to definitively confirm the genetic status of the HM bison. Given the Department of Interior initiative to conserve and restore wild bison [23] and with the HM bison herd occurring within one of the most important conservation priority areas of the roadless BLM lands in the western USA [24], further genetic screening of this herd has been a priority for some time [9]. Additionally, genetic information is needed for planning the future involvement of HM bison in metapopulation management across this species’ range [13].

With 129 available genetic samples for this herd, our objectives were to assess the overall genetic variation as it may relate to fitness and potential inbreeding effects on the HM bison herd by: (1) testing for mitochondrial and nuclear introgression; (2) assessing genetic diversity (observed heterozygosity and average alleles per locus) in the HM herd in comparison to the 8 largest US federal herds; (3) determining the genetic ancestry contribution from the 8 federal herds to the HM herd; and finally (4) determining the genetic relationship of the HM herd with the 8 federal herds.

## Materials and Methods

### Sample collection

Tail hair samples were pulled from 86 HM bison during helicopter capture and collar operations between January 2011 and January 2013 as part of a larger research effort on the HM bison herd. Every effort was taken to ensure even sample distribution from across the entire population (Fig 1). Bison were released immediately after collaring and sample collection. Samples were stored dry in coin envelopes at room temperature until analysis in 2014. An additional 22 samples collected in 2004 from hunter-killed animals, but not yet analyzed, were combined with a further 21 samples from previous exploratory work on the HM herd [9,20], bringing the total sample size to 129 bison. Samples were collected in accordance with Utah State University Institutional Animal Care and Use Committee approved protocol #1452, and the Utah Division of Wildlife Resources Certificate of Registrations for Banding, Collection, and Salvage #6BANC8393 from public land, with permission from the Bureau of Land Management and did not involve endangered or protected species.

#### PCR conditions and primer multiplexes

Mitochondrial primers (16S and TPW) and assay used for genotyping for the presence of domestic cattle mitochondrial DNA were described by Ward et al. 1999 [20]; in which amplification at the TPW mitochondrial marker indicates the presence of domestic cattle mitochondrial DNA and amplification of the 16S and no amplification of the TPW mitochondrial markers indicates bison mitochondrial DNA. Mitochondrial DNA is maternally inherited without recombination, making the mitochondrial test useful in assessing domestic cattle introgression in the maternal lineage of the HM bison [20]. We ran a cattle-positive control with the HM bison samples during the PCR step to ensure the amplification of the TPW marker for domestic cattle mitochondrial DNA. If the positive control amplified, then the TPW marker would have also amplified in any HM samples that had the domestic cattle mitochondrial type (if any).

Multiplexed primer mixes were used for genotyping nuclear microsatellites, as described for previous related studies [9,25,26]. The 14 primer pairs used to assess introgression for this analysis were previously evaluated for domestic cattle and bison origin by comparing alleles found to be in 84 wood bison and 328 plains bison. These were confirmed to provide a reliable assessment of past introgression from cattle—bison hybridization using 3,301 bison samples and 64 cattle samples from 5 domestic cattle breeds [9,27]. We followed the same established protocols [9,27]. An additional 26 microsatellite loci were used to assess genetic diversity. A list of the microsatellites used can be found in supporting material (S1 Table). Previously collected and published data for the 8 DOI herds were used for the comparison of genetic diversity and relationships to HM [9,27,28]

PCR conditions and primer multiplexes consisted of 5 μL total volume with: 1 μL of DNA (extracted from hair follicles described by KAPA Express Extraction Kits, KapaBiosystems); 0.05 to 0.4 μM of each primer (40 nuclear primer pairs and 2 mitochondrial primer pairs); 1 x MasterAmp PCR enhancer (Epicentre, Madison, Wisconsin); 500 μM deoxynucleotide triphosphates; 3.0 mM MgCl_2_; 1 x reaction buffer; 0.5 units *Taq* DNA polymerase (Promega, Madison, Wisconsin). PCR products were separated on an ABI 3130 genetic analyzer (Applied Biosystems, Foster City, California) using an internal size standard (Mapmarker 400 and 1000, Bioventures, Inc., Murfreesboro, Tennessee). GeneMapper 3.7 software (Applied Biosystems) was used for allele identification and comparison.

#### Data analysis

Accompanying the 2 mitochondrial markers described above, nuclear introgression of domestic cattle DNA was evaluated for the HM plains bison samples using 14 nuclear polymorphic microsatellite markers to identify the presence domestic cattle chromosome regions in bison [9]. In addition to the 14 nuclear polymorphic microsatellite markers used for the detection of domestic cattle introgression, all 129 samples from the HM bison herd were genotyped for 26 polymorphic nuclear microsatellite markers that were previously shown to evaluate inter-population dynamics among bison herds [25], resulting in a total of 40 nuclear polymorphic microsatellite markers and 2 mitochondrial markers. Full details on the markers used can be found in references 20 and 25. Excel Microsatellite Toolkit [29] was used to determine values for heterozygosity, average number of alleles per locus, and format files for further downstream analysis for all 26 loci. Allelic richness for each population at all 26 loci was obtained using FSTAT 2.9.3.2 [30]. This information was used to determine inter-population dynamics in the HM samples and were compared to our published genetic diversities of 8 federal Department of Interior (DOI) bison herds [9] using a two-tailed t-tests. Combining the 26 polymorphic nuclear markers used for inter-population dynamics and the 14 polymorphic nuclear markers (i.e., 40 total microsatellite loci) used to detect nuclear introgression of domestic cattle, the relationship of HM to the 8 DOI herds was assessed using the multilocus Bayesian clustering method across 10 iterations in the Structure 2.1 software, using the 8 DOI herds as known (defined) populations (K = 8) [9,28,31]. K was set to 8 for the defined populations based on previous experiments and publications in the lab using the same data set for the DOI herds [28]. Individual and population assignments within each iteration were sorted and aligned using CLUMPP 1.0 [32], and subsequent assignments were visualized using DISTRUCT [33].

### Relationships among herds

Excel Microsatellite Toolkit [29] was also used to format the master genotype file to be read by FSTAT 2.9.3.2 [30] to produce allele frequencies per locus within each population. A formatted file of allele frequencies per locus and population was read by contml.exe in the PHYLIP 3.695 [34] package to create an unrooted tree diagram using the maximum likelihood method in FigTree v. 1.4.2 (http://tree.bio.ed.ac.uk/software/figtree/), comparing the relationships of the HM population with the 8 DOI herds.

## Results

Amplification at the TPW marker would indicate the domestic cattle mitochondrial type, but all 129 plains bison samples from the HM showed no evidence of that. All of the HM samples had amplification only at the 16S marker, indicating that they only contain bison mitochondrial DNA. The known cattle controls amplified for TPW only, with no amplification of 16S. Alleles that are known to occur in cattle at the 14 nuclear markers [9,28] were not detected in HM bison and support previous findings for bison herds with no nuclear domestic cattle introgression detected (Table 1). Genotypes for the 40 nuclear markers for each HM sample can be found in the supporting material (S2 Table).

**Table 1:** Genetic Diversity and Domestic Cattle Introgression.

Population	Sample Size	Loci Typed	Observed Heterozygosity	Average Allelic Richness	Average Alleles Per Locus	Domestic Cattle MtDNA	Domestic Cattle Nuclear Markers And Frequencies (if detected)
**Badlands National Park**	328	26	0.592	4.17*	4.46	none detected	BM4307, 197-bp allele, .1355; BMS2270, 94-bp allele, .0315
**Fort Niobrara NWR**	178	26	0.607	4.11*	4.42	none detected	BM4307, 197-bp allele, .1348
**National Bison Range**	179	26	0.632*	4.62*	4.92	0.0187	BM7145, 116-bp allele, .0383
**Theodore Roosevelt NP—North**	309	26	0.572	3.45	3.62	none detected	BM4307, 197-bp allele, .1626
**Theodore Roosevelt NP—South**	368	26	0.585	4.10*	4.35	none detected	BM4307, 197-bp allele, .1151
**Wind Cave NP**	345	26	0.643*	4.55*	4.81	none detected	none detected
**Wichita Mountains NWR**	37	26	0.564	4.09	4.12	none detected	BM1314, 157-bp allele, .0901
**Yellowstone National Park**	505	26	0.603*	4.26*	4.62	none detected	none detected
**Henry Mountains**	129	26	0.554	3.57	3.88	none detected	none detected

The HM samples had moderate genetic diversity as indicated by mean (± SD) observed heterozygosity (55.4% ± 0.009%) and alleles per locus (3.88 ± 1.21; Table 1), when compared to the 8 DOI herds. The HM bison population had statistically lower observed heterozygosity than 3 of the 8 DOI herds (NBR, WCNP and YNP). Average allelic richness in the HM herd was significantly different than 6 of the 8 herds, with only Theodore Roosevelt NP-North having a lower average allelic richness.

Genetic diversity for 26 microsatellite loci and cattle DNA introgression for 8 bison populations [9] conserved by the US federal government (DOI) presented for comparison with the Henry Mountains population. Observed heterozygosity and average allelic richness values marked with an * are significantly different (p<0.05) than the Henry Mountains value using a two-tailed *t*-test. DOI results were collected and published by our lab [9].

**Fig 2:**
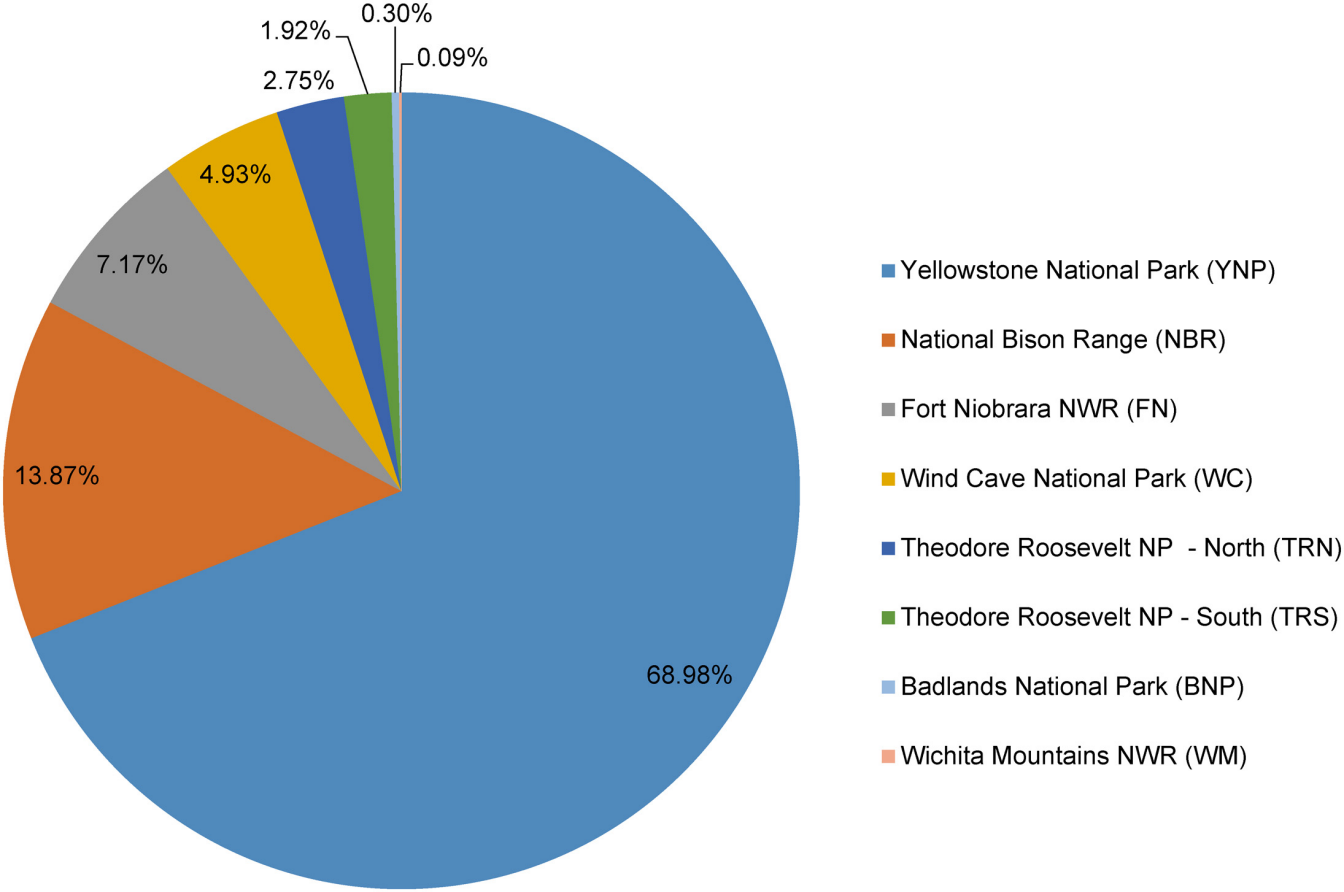
Genomic Contributions. Genomic contributions of 8 US federal bison herds to the Henry Mountains herd, in which 129 animals were sampled for 40 microsatellite loci. Herds were identified *a priori* for analysis. Contributions of <10% were considered insignificant.

**Fig 3:**
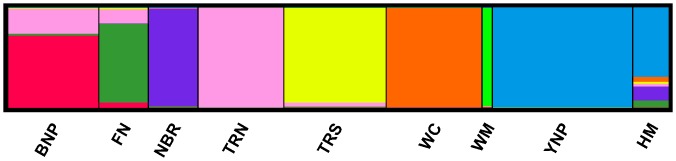
Histogram of Genomic Contributions. A histogram showing the relative contributions of each of the 8 federal bison herds to the Henry Mountains herd for 40 microsatellite loci, in which 129 animals were sampled with K = 8. It also shows contributions of the 8 federal herds to each other’s genetic composition. Herds were identified *a priori* for analysis. See Fig 2 for herd name abbreviations.

Population assignments by Structure 2.1 software were sorted and aligned in CLUMPP to determine genetic contributions of each of the 8 DOI herds to the HM population. The Structure analysis for genetic contribution and ancestry of the HM population was found to have significant genomic contributions from YNP (approximately 69%) and National Bison Range (NBR, approximately14%; Figs 2 and 3) and less than 10% for the remaining 6 DOI herds, indicating that the genetic makeup of the HM bison is most similar to the YNP and NBR populations. A histogram showing the relative contributions of each of the 8 US federal bison herds for each individual HM sample can be found in the supporting information (S1 Fig).

**Fig 4:**
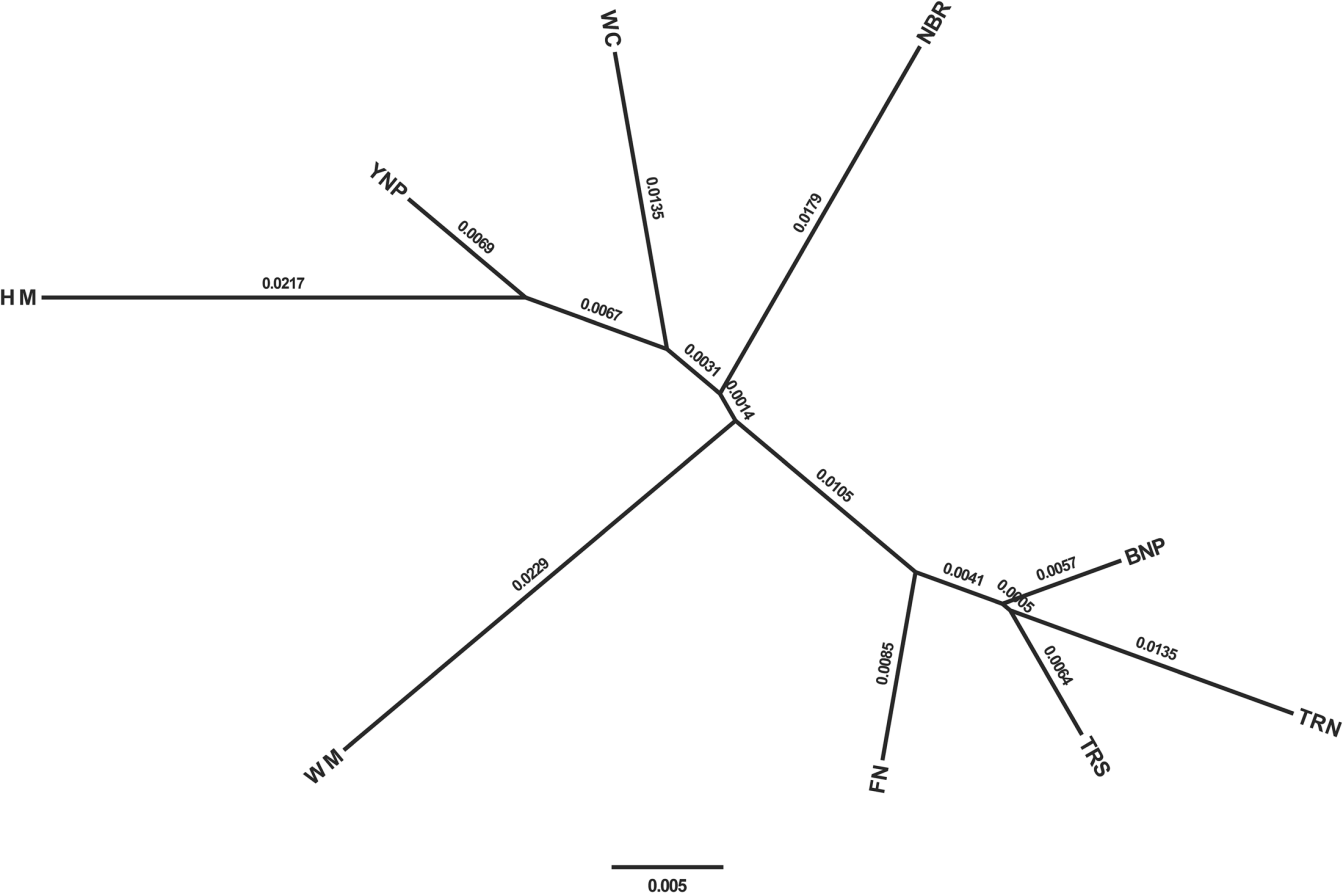
Dendrogram. Unrooted tree diagram with maximum likelihood scores comparing Henry Mountain bison samples to 8 US federal herds using 40 polymorphic loci. See Fig 2 for herd name abbreviations.

The sources of genetic contributions from the 8 DOI herds were also analyzed individually for the HM samples (S3 Table). Any source population with <10% genomic contribution was considered negligible and was removed from the analysis. The sources of genetic contributions at the individual level match those at the population level, with major contributions to HM from only YNP and the NBR. Based on the 40 polymorphic markers, an unrooted ML dendogram was constructed using the allele frequencies within each population and with maximum likelihood scores showing the amount of expected accumulated variance between each population. As expected, and confirming the STRUCTURE analysis completed, the HM samples were found to cluster closest to YNP (Fig 4). The Structure analysis and the ML dendogram suggested slightly different placements of the DOI herds relative to HM, which is not uncommon with different statistical analyses. The Structure analysis had NBR as the second genomic contributor to HM after YNP, whereas the ML dendogram placed Wind Cave (WC) in-between HM and NBR.

## Discussion

The ability to identify bison herds without domestic cattle introgression is important for conserving the original bison genome and also for providing founder animals with unimpaired fitness. There is, for example, an association between mitochondrial DNA type (bison or cattle) and body size, which is likely deleterious in this species with its highly competitive mating system. In both nutritionally rich and poor environments, bison with domestic cattle mitochondrial DNA are on average smaller than bison with bison mitochondrial DNA [35], demonstrating at least one of the possibly numerous phenotypic expressions of genetic introgression that could be deleterious. It is, however, possible that introgression could provide increased fitness in the form of adaptive introgression [36], though this has not been demonstrated in bison.

Our assessment of the genetic status of the HM bison herd found no evidence of introgression of cattle DNA in either mitochondrial or nuclear genomes (Objective 1). This does not ensure that the HM bison herd is free of domestic cattle introgression, but rather we were not able to detect the presence of domestic cattle genetics based on this technology. This is, however, extremely encouraging as the HM herd now joins Wind Cave and Yellowstone National Parks as the only US publically owned conservation herds in which cattle introgression has not been detected or surmised based on herd history. This is notable because the HM bison have shared the HM rangeland with cattle for >70 years with no detectable hybridization.

Moderate levels of genetic diversity (observed heterozygosity) were detected in the HM herd (Objective 2) in comparison to the 8 DOI federal bison herds (Table 1). In relation to those herds the observed heterozygosity of the HM samples was the lowest, though not significantly different than all but WC, YNP, and NBR. This is not altogether unexpected given the small number of founders. Despite this, the genetic diversity detected confirms that the HM bison herd grew quickly enough from its founding bottleneck to escape the negative fitness effects of inbreeding. The Texas State Bison Herd (TSBH) at Caprock Canyons State Park provides an example of dangerously low levels of genetic diversity that were contributing to unusually high frequencies of stillborn calves and spontaneous of abortions. When compared with the 8 federal bison herds, the TSBH genetic diversity, calculated using the same microsatellite loci as this study, was the lowest with averages of 0.399 observed heterozygosity and 2.5 alleles per locus [26]. This low diversity was predicted to cause extinction of the TSBH in 50 years without genetic rescue [26], but the situation has improved with the introduction of some breeding bulls from other herds. A moderate level of approximately 0.439 observed heterozygosity and an average of 3.46 alleles per locus had been attained in the TSBH as of 2013 (Dobson et al. unpublished), though these are still lower than the values found in the HM. Considering the average number of alleles per locus, the HM bison herd ranked just above the Theodore Roosevelt National Park—North herd, or second-lowest among the US federal bison herds (Table 1).

We found that the HM herd is primarily related to and descended from the YNP source population (Objectives 3 and 4), as was expected from the existing management and herd history. There were also some genetic contributions from the National Bison Range, from where 18 females are known to have been introduced into YNP before the HM translocation took place [2]. These findings confirm that the HM bison herd represents a genetically important subpopulation of the YNP-based metapopulation. It meets the YNP standard of no detectable cattle introgression but is also free of the disease (brucellosis) issues prominent in the YNP herd. Furthermore, with these findings, we now have a third US bison herd to consider as a source of introgression-free bison to help establish new subpopulations across the former range of the species [13]. Care should be taken to ensure that the HM bison remain isolated from all other herds except YNP (with confirmed brucellosis-free bison only) and Windcave NP, where cattle introgression screening has also been negative. Additionally, current advances with the completion of the bison reference genome will further our understanding of the genetic status of the HM bison herd and other conservation herds across North America [37]

Taken together, our analyses of these129 individuals indicate that the free-ranging bison in the Henry Mountains of Utah are genetically diverse, have no detected introgression of domestic cattle DNA, and are descended from a mixture of 2 federal bison herds with the majority being from Yellowstone National Park. Consequently, as the only demonstrated introgression free, disease-free, and free-ranging bison population in North America, we propose that the Henry Mountains should now be recognized as a primary source for ongoing conservation of the North American plains bison.

## Supporting Information

S1 Fig Histogram of Genomic Contributions for each individual HM sample. A histogram showing the relative contributions of each of the 8 federal bison herds to each individual Henry Mountains sample. It also shows contributions of the 8 federal herds to each individual samples genetic composition. See Fig 2 for herd name abbreviations.
(PDF)

S1 Table Loci Information. Information for 40 microsatellite loci used in this study. (DOCX)

S2 Table Individual genotypes. The individual genotypes for each sample and locus.
(XLSX)

S3 Table Individual genetic contributions of 8 core U.S. federal herds to the HM samples. Contributions of less than 10% are considered insignificant and were not shown for those populations.
(DOCX)
