# Supplementary figures and images for: Genetic Analysis of the Henry Mountains Bison Herd
Source: PLoS One. 2015 Dec 16;10(12):e0144239. doi: 10.1371/journal.pone.0144239 (PMC4682953; doi:10.1371/journal.pone.0144239)

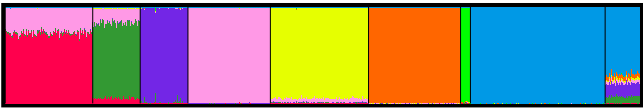

*BNP*

*FN*

*NBR*

*TRN*

*TRS*

*WC*

*WM*

*YAP*

*YM*

Supplement: S1 Fig — A histogram showing the relative contributions of each of the 8 federal bison herds to each individual Henry Mountains sample. It also shows contributions of the 8 federal herds to each individual samples genetic composition. See Fig 2 for herd name abbreviations. (PDF) [file pone.0144239.s001.pdf]
