# Supplementary material for: Genetic Analysis of the Henry Mountains Bison Herd
Source: PLoS One. 2015 Dec 16;10(12):e0144239. doi: 10.1371/journal.pone.0144239 (PMC4682953; doi:10.1371/journal.pone.0144239)
Supplement: S1 Table — (DOCX) [file pone.0144239.s002.docx]

**S1 Table.** Information for 40 nuclear microsatellite loci used in this study

| **Locus** |
| --- |
|  |
| AGLA17 |
| AGLA293 |
| BL1036 |
| BM1225 |
| BM1314 |
| BM1706 |
| BM17132 |
| BM1862 |
| BM1905 |
| BM2113 |
| BM4107 |
| BM4307 |
| BM4311 |
| BM4440 |
| BM4513 |
| BM47 |
| BM6017 |
| BM711 |
| BM7145 |
| BM720 |
| BMS1001 |
| BMS1074 |
| BMS1315 |
| BMS1675 |
| BMS1716 |
| BMS1857 |
| BMS2270 |
| BMS4040 |
| BMS410 |
| BMS510 |
| BMS527 |
| CSSM36 |
| CSSM42 |
| HUJ246 |
| ILSTS102 |
| INRA189 |
| RM185 |
| RM372 |
| RM500 |
| SPS113 |
| TGLA122 |
| TGLA227 |
