# Supplementary material for: Genetic Analysis of the Henry Mountains Bison Herd
Source: PLoS One. 2015 Dec 16;10(12):e0144239. doi: 10.1371/journal.pone.0144239 (PMC4682953; doi:10.1371/journal.pone.0144239)
Supplement: S3 Table — Contributions of less than 10% are considered insignificant and were not shown for those populations. (DOCX) [file pone.0144239.s004.docx]

**S3 Table.** **Individual genetic contributions of 8 core U.S. federal herds to the HM samples.** Contributions of less than 10% are considered insignificant and were not shown for those populations.

| **Sample** | **National Bison Range** | **Yellowstone National Park** |
| --- | --- | --- |
| 2014004356 | 0.1238 | 0.7193 |
| 2014004357 | 0.1452 | 0.7096 |
| 2014004358 | 0.1417 | 0.6894 |
| 2014004359 | 0.1297 | 0.7119 |
| 2014004360 | 0.1312 | 0.7259 |
| 2014004361 | 0.1284 | 0.7344 |
| 2014004362 | 0.1444 | 0.7053 |
| 2014004363 | 0.1323 | 0.7067 |
| 2014004364 | 0.1544 | 0.6993 |
| 2014004365 | 0.1375 | 0.7193 |
| 2014004366 | 0.1575 | 0.6815 |
| 2014004367 | 0.1347 | 0.6939 |
| 2014004368 | 0.1233 | 0.7324 |
| 2014004369 | 0.136 | 0.7228 |
| 2014004370 | 0.1672 | 0.6757 |
| 2014004371 | 0.1408 | 0.6969 |
| 2014004372 | 0.1392 | 0.721 |
| 2014004373 | 0.1206 | 0.7194 |
| 2014004374 | 0.1321 | 0.7227 |
| 2014004375 | 0.146 | 0.6851 |
| 2014004376 | 0.1309 | 0.7091 |
| 2014004377 | 0.1331 | 0.6873 |
| 2014004378 | 0.139 | 0.7055 |
| 2014004379 | 0.1271 | 0.7038 |
| 2014004380 | 0.1394 | 0.6949 |
| 2014004381 | 0.1378 | 0.6634 |
| 2014004382 | 0.141 | 0.6984 |
| 2014004383 | 0.1282 | 0.7166 |
| 2014004384 | 0.1507 | 0.7019 |
| 2014004385 | 0.1251 | 0.7217 |
| 2014004386 | 0.1232 | 0.7245 |
| 2014004387 | 0.1456 | 0.7049 |
| 2014004388 | 0.1207 | 0.7244 |
| 2014004389 | 0.1516 | 0.6848 |
| 2014004390 | 0.1316 | 0.7132 |
| 2014004391 | 0.1157 | 0.7131 |
| 2014004392 | 0.1578 | 0.6544 |
| 2014004393 | 0.1359 | 0.7187 |
| 2014004394 | 0.1296 | 0.7154 |
| 2014004395 | 0.1346 | 0.699 |
| 2014004396 | 0.1517 | 0.6697 |
| 2014004397 | 0.1503 | 0.6744 |
| 2014004398 | 0.1307 | 0.6805 |
| 2014004399 | 0.1298 | 0.7002 |
| 2014004400 | 0.1504 | 0.7062 |
| 2014004401 | 0.1396 | 0.686 |
| 2014004402 | 0.1508 | 0.6845 |
| 2014004403 | 0.1418 | 0.6845 |
| 2014004404 | 0.1273 | 0.7155 |
| 2014004405 | 0.1544 | 0.6869 |
| 2014004406 | 0.1362 | 0.6773 |
| 2014004407 | 0.1332 | 0.7159 |
| 2014004408 | 0.1362 | 0.694 |
| 2014004409 | 0.1245 | 0.6929 |
| 2014004410 | 0.1388 | 0.6748 |
| 2014004411 | 0.1324 | 0.671 |
| 2014004412 | 0.1653 | 0.6608 |
| 2014004413 | 0.1529 | 0.6458 |
| 2014004414 | 0.1279 | 0.7041 |
| 2014004415 | 0.1369 | 0.6843 |
| 2014004416 | 0.1339 | 0.6779 |
| 2014004417 | 0.1523 | 0.677 |
| 2014004418 | 0.1261 | 0.6995 |
| 2014004419 | 0.1343 | 0.7002 |
| 2014004420 | 0.1295 | 0.7035 |
| 2014004421 | 0.1392 | 0.6592 |
| 2014004422 | 0.1332 | 0.6854 |
| 2014004423 | 0.1276 | 0.7028 |
| 2014004424 | 0.1402 | 0.6836 |
| 2014004425 | 0.1507 | 0.6962 |
| 2014004426 | 0.1225 | 0.6984 |
| 2014004427 | 0.1367 | 0.6911 |
| 2014004428 | 0.1357 | 0.6818 |
| 2014004429 | 0.127 | 0.6991 |
| 2014004430 | 0.1265 | 0.7028 |
| 2014004431 | 0.1246 | 0.7224 |
| 2014004432 | 0.1259 | 0.6931 |
| 2014004433 | 0.1311 | 0.712 |
| 2014004434 | 0.1293 | 0.6804 |
| 2014004435 | 0.1275 | 0.7048 |
| 2014004436 | 0.1429 | 0.6898 |
| 2014004437 | 0.143 | 0.6969 |
| 2014004438 | 0.1324 | 0.7043 |
| 2014004439 | 0.1441 | 0.6819 |
| 2014004440 | 0.1252 | 0.6891 |
| 2014004441 | 0.1413 | 0.6786 |
| 263 | 0.1327 | 0.6698 |
| 264 | 0.152 | 0.6716 |
| 265 | 0.1319 | 0.6643 |
| 266 | 0.1529 | 0.69 |
| 267 | 0.1393 | 0.6877 |
| 268 | 0.1626 | 0.6484 |
| 269 | 0.1382 | 0.7141 |
| 270 | 0.1413 | 0.7105 |
| 271 | 0.1412 | 0.6913 |
| 272 | 0.1412 | 0.6911 |
| 273 | 0.1464 | 0.6856 |
| 274 | 0.1182 | 0.7115 |
| 275 | 0.1305 | 0.6919 |
| 276 | 0.1419 | 0.6721 |
| 277 | 0.1577 | 0.6518 |
| 278 | 0.1525 | 0.673 |
| 279 | 0.1684 | 0.6292 |
| 280 | 0.1657 | 0.6509 |
| 281 | 0.1475 | 0.6403 |
| 282 | 0.1224 | 0.7111 |
| 283 | 0.138 | 0.6827 |
| 34153 | 0.1317 | 0.6879 |
| 34154 | 0.1336 | 0.6877 |
| 34155 | 0.1488 | 0.6674 |
| 34156 | 0.157 | 0.6447 |
| 34157 | 0.134 | 0.6793 |
| 34158 | 0.1612 | 0.655 |
| 34159 | 0.1486 | 0.6322 |
| 34160 | 0.1379 | 0.6962 |
| 34161 | 0.1453 | 0.6786 |
| 34162 | 0.1213 | 0.6999 |
| 34163 | 0.1312 | 0.6932 |
| 34164 | 0.1525 | 0.6578 |
| 34165 | 0.1351 | 0.6806 |
| 34166 | 0.1148 | 0.7062 |
| 34167 | 0.1504 | 0.6482 |
| 34168 | 0.1416 | 0.692 |
| 34169 | 0.1401 | 0.6767 |
| 34170 | 0.1458 | 0.6714 |
| 34171 | 0.1382 | 0.6794 |
| 34172 | 0.1364 | 0.6421 |
| 34173 | 0.1568 | 0.6296 |
| 34174 | 0.1388 | 0.7094 |
